# Capsaicin and Gut Microbiota in Health and Disease

**DOI:** 10.3390/molecules25235681

**Published:** 2020-12-02

**Authors:** Adrian Eugen Rosca, Mara Ioana Iesanu, Carmen Denise Mihaela Zahiu, Suzana Elena Voiculescu, Alexandru Catalin Paslaru, Ana-Maria Zagrean

**Affiliations:** 1 Division of Physiology and Neuroscience, Department of Functional Sciences, “Carol Davila” University of Medicine and Pharmacy, 050474 Bucharest, Romania; mara-ioana.iesanu@rez.umfcd.ro (M.I.I.); carmen.zahiu@umfcd.ro (C.D.M.Z.); suzana.voiculescu@umfcd.ro (S.E.V.); catalin.paslaru@umfcd.ro (A.C.P.); 2 Department of Cardiology, Emergency University Hospital of Bucharest, 050474 Bucharest, Romania; 3 Department of Pediatric Gastroenterology, “Marie Curie” Children’s Clinical Hospital, 041434 Bucharest, Romania

**Keywords:** capsaicin, capsinoids, microbiota, antioxidant, antitumoral, energy metabolism, immune system, diabetes, obesity, inflammatory bowel disease

## Abstract

Capsaicin is a widespread spice known for its analgesic qualities. Although a comprehensive body of evidence suggests pleiotropic benefits of capsaicin, including anti-inflammatory, antioxidant, anti-proliferative, metabolic, or cardioprotective effects, it is frequently avoided due to reported digestive side-effects. As the gut bacterial profile is strongly linked to diet and capsaicin displays modulatory effects on gut microbiota, a new hypothesis has recently emerged about its possible applicability against widespread pathologies, such as metabolic and inflammatory diseases. The present review explores the capsaicin–microbiota crosstalk and capsaicin effect on dysbiosis, and illustrates the intimate mechanisms that underlie its action in preventing the onset or development of pathologies like obesity, diabetes, or inflammatory bowel diseases. A possible antimicrobial property of capsaicin, mediated by the beneficial alteration of microbiota, is also discussed. However, as data are coming mostly from experimental models, caution is needed in translating these findings to humans.

## 1. Introduction

Capsaicin (CAP) is a compound found in almost all types of peppers, responsible for their characteristic pungent aroma. CAP was first described in 1816 by Christian Bucholz and its chemical composition, 8-methyl-N-vanillyl-6-nonenamide, was revealed in 1919 [1]. CAP binds to a transient receptor potential channel of vanilloid subtype 1 (TRPV1) found on A- and C-delta fibers in the nociceptive sensory pathway, which initiates the signal transduction cascade that finally leads to desensitization of the afferent nerve fibers [1,2].

CAP from the berries of *Capsicum* species is among the most widespread spices used in cuisines throughout the world [3], and harbors many benefits that have extensively been documented in various in vivo, ex vivo, and in vitro studies. CAP and the related nonpungent capsinoids (capsiate, dihydrocapsiate, nordihydrocapsiate) have been proven to elicit analgesic, antioxidant, anti-inflammatory, anticarcinogenic, weight modulatory, cardio-protective, anti-lithogenic, and circadian-modulatory effects [4]. Therefore, besides its culinary utilization, CAP has been used as a therapeutic agent in various painful chronic conditions, such as those encountered in diabetic and nondiabetic neuropathy, temporo-mandibular joint disorder, burning mouth syndrome, postherpetic neuralgia, osteoarthritis, or rheumatoid arthritis [1,5,6,7]. On the other hand, there are data showing that chronic exposure to high doses of CAP can enhance the development of liver, stomach, duodenal, and prostate cancer, and can induce peptic ulcers [1,8,9]. Even if several adverse effects have been described on common doses, CAP has no absolute contraindication, and only a few relative contraindications, such as asthma. The antidote is still unknown, but there were no reported overdoses of any preparation of capsaicin [1]. Moreover, one prospective study noted that subjects who consumed spicy food almost daily had a 14% lower risk of death, without being able to make a causal relationship [10].

Changing the biodiversity of gut flora has been associated with a high risk of autoimmune and allergic diseases, obesity, inflammatory bowel disease (IBD), diabetes, cancer, cardiovascular diseases, and cirrhosis [11,12,13,14,15,16,17,18,19,20]. Thus, remodeling the gut microbiome by dietary supplements or food additives could represent an innovative therapeutic strategy against various diseases. Spicy food, especially CAP, recently drew considerable attention from the perspective of their positive action on gut flora, by eliminating the disease-causing enteric pathogens, and encouraging the growth of beneficial bacteria [21,22,23]. However, due to frequent consumption and its therapeutic valence, a comprehensive assessment of CAP effects is an important goal from the public health standpoint [8].

Considering the increasing interest in gut flora regulation, and the emerging data linking CAP and capsinoids to the gut microbiota composition, abundance, and function, we aimed in this review to systematize these effects and to underline the possible mechanisms by which CAP exerts its influence.

## 2. Capsaicin and Its Systemic and Local Effects

CAP has multiple actions on the body that are mediated by a variety of molecular pathways. The anti-inflammatory capacity of CAP has been demonstrated and multiple mechanisms are involved, such as the inhibition of pro-inflammatory substances IL-6, TNF-alpha, PGE2, and nitric oxide production [24]. In a rat model of sepsis, it has been shown that a small dose of CAP (1 mg/kg) administered subcutaneously prevented the production of proinflammatory cytokines and increased plasma levels of IL-10, while higher doses (150 mg/kg) did not exert the same effect [25]. The antioxidative effect of CAP has been extensively studied ex vivo, showing that it has a higher antioxidative power than melatonin [26]. The free radical scavenging ability has been studied in aqueous and lipid solutions, indicating CAP as a powerful antioxidative substance [27]. However, there are only a few in vivo studies that assess its antioxidative effect in biological systems. Results from various studies suggest a significant protective effect of CAP against oxidative stress, by enhancing FRAP (the ferric reducing antioxidant power), GSH level, PMRS activity (plasma membrane redox system), and ameliorating reactive oxygen species (ROS), MDA (malondialdehyde), and AOPP (advanced oxidation protein products) in the plasma [28].

CAP is a highly selective and potent exogenous agonist for the TRPV1 receptor, a transmembrane ion channel that provides complex responses to temperature, pH, and endogenous lipids [29]. Endogenous and exogenous activation of TRPV1 initiates sodium and calcium ions-mediated depolarization. Nociceptive nerve endings that express TRPV1 (C- and some Aδ-fibres) initiate action potentials, which are sent into the central nervous system, leading to a subjective sensation of warming, and burning. The CAP-related activation of TRPV1 can generate a different, more persistent effect, in contrast with the transient effect depicted earlier [30]. Skin-applied topical CAP lowers cutaneous hypersensitivity, changes cutaneous blood flow [31], and reduces pain by a process called “defunctionalization” of nociceptor fibers [32,33]; thus, CAP has been used as an analgesic substance since the early 1980s [2]. 

There is strong experimental evidence that CAP provides anticarcinogenic effects, by inhibiting tumor initiation, development, progression, and metastasis formation [34]. It has been shown that CAP modulates the activity of genes involved in cancer cell life span, angiogenesis, and metastasis [35]. Anticarcinogenic pathways also include cell cycle arrest and apoptosis. A recent review suggests that CAP induces apoptosis in 40 different cancer cell lineages, including prostate, liver, bladder, skin, leukemic cells, lung, colon, and endothelial cells [9]. Its pro-apoptotic activity appears to be mediated also by TRPV1 receptor activation [36]. Studies on pancreatic cells correlate CAP-related apoptosis with ROS production, c-Jun N-terminal Kinase (JNK) activation, mitochondrial membrane functional changes, cytosolic cytochrome C release, and caspase cascade initiation [37]. CAP acts as an antagonist coenzyme and blocks plasmalemmal NADPH oxidase [38], arrests cell cycles in bladder carcinoma cells by inhibiting CDK2, CDK4, and CDK6 [39], and initiates apoptosis in tumoral cells by activation of the p53 tumor-suppressor unit [40].

CAP modulates the energy balance and is involved in obesity prevention and treatment by multiple mechanisms. In human studies, it has been shown that CAP exhibits anorexigenic sensations, such as satiety, when added to the diet [41,42]. It also decreases food intake, suppresses the desire to eat, and hunger. The mechanisms are not fully understood, but one plausible theory is that CAP ingestion increases sympathetic nervous system activity, as catecholamines have an anorexigenic effect [41]. CAP ingestion also increases glucagon-like peptide 1 secretion, an anorexigenic hormone, decreases orexigenic hormone secretion ghrelin in human subjects [43], and modulates adipogenesis. This information will be detailed in the next sections in the light of microbiota composition modulation.

CAP-sensitive nerves are widely present in the cardiovascular system and act through substance P or calcitonin gene-related peptide (CGRP) pathways. CGRP is a potent vasodilator and regulates blood pressure under physiological and pathological conditions [44]. CAP stimulates the release of CGRP via TRPV1 and decreases blood pressure [45,46]. Studies have shown that CAP also has an important antiatherogenic effect, by prolonged activation of TRPV1, leading to decreased lipid storage and improving atherosclerotic lesions in the mouse aorta [47]. CAP inhibits platelet aggregation [48], and by this, it provides protection against cardiovascular diseases [49]. 

The present review explores CAP involvement in the physiopathology of the intestinal microbiome. 

## 3. Role of Gut Microbiota in Health and Disease

The gastrointestinal tract harbors a variety of microorganisms called the gut microbiota. There are approximately 100 trillion microorganisms in an adult human microbiota, which weigh up to 2 kg. The majority of the intestinal bacteria fall into two bacterial phylotypes, *Firmicutes* and *Bacteroidetes*, which gather more than 90% of the total community of microorganisms [50].

The intestinal microbiome describes the collective genome of the bacterial communities that reside in the gut [51]. Metagenomics sequencing provides the opportunity to identify the bacterial constellations at the genus and family level. It has been proven that the human microbiome has a common gene set, shared by almost half of the individuals of the studied cohort, considered the core microbiome that provides a core set of functions [52]. Based on the Kyoto Encyclopedia of Genes and Genomes, Qin et al. identified two main types of functions of the microbiome, one for “house-keeping,” necessary for all bacteria, and one specific function for the gut. In the core microbiome, there are genes involved in the fermentation of dietary or intestinal complex sugars, their conversion into short-chain fatty acids (SCFA), and the synthesis of vitamins such as vitamin K or biotin.

A great variety usually characterizes a healthy intestinal flora, and there are several indices used to estimate within- and between-different-sample diversity. Whittaker was the first to describe two of the most used indices for measuring biodiversity. Alpha diversity describes the variety within a sample such as within the human gut, while beta diversity estimates the degree to which samples may differ from one another [53]. 

Early life provides a window of opportunity during which the composition and function of the microbiota can be altered. The colonization of a newborn begins before birth, during the intrauterine period, which was recently proven by identifying microbes in the placenta [54]. The bacterial communities can be influenced by many factors such as mode of delivery and nutritional provision in early life, drugs, especially antibiotic therapy, and early-life stress [55]. Moreover, the first 1000 days from the conception moment until 2 years of age are crucial for the proper development of the host, and the adult-like complexity of the gut flora is attained by the end of this period.

After this period, the microbiota undergoes continuous change, its diversity and composition being modified by numerous factors such as diet and lifestyle, drugs, stress, infections, and aging. The gut microbiota diversity decreases in the elderly [56], without having a clear pattern of the bacterial species change by aging. The findings regarding gut microbiota composition in the elderly were inconsistent, suggesting that it is correlated with the diet rather than with the age [56,57,58].

The gut microbes have a tremendous potential to influence human health. Although the assumption that the microbiome represents a pivotal factor in human health is not novel [59], the intestinal microenvironment has only recently gained attention.

First, the microbiota, through stimulation of epithelial cells regeneration and mucus layer production, acts as an intestinal barrier against the entry of viruses, bacteria, and parasites. The bacterial communities’ metabolism is predominantly anaerobic. Its end-products are mainly SCFAs, such as acetate, propionate, and butyric acid, that serve not only as energy substrates for the intestinal epithelial cells, but also as signaling molecules between the gut and other organs [60]. SCFAs activate receptors in the gut, adipose tissue, immune system, bone marrow, pancreas, heart, and skeletal muscles, known as the FFA2 and FF3 receptors (free fatty acid receptors), or as orphan G-protein-coupled receptors GPR43 and GPR41 [61,62,63]. Acetate and propionate modulate appetite and satiety [64,65], whereas butyrate and propionate regulate energy metabolism through gut hormones [66]. Furthermore, acetate is important for the survival and growth of the gut major butyrogenic bacterial species, *Faecalibacterium prausnitzii* or *Roseburia intestinalis/Eubacterium rectale* [67].

A considerable body of evidence links gut microbiota to immune system maturation. A causal relationship between bacterial colonization and host immunity is strongly supported by studies on germ-free animal models. Healthy bacterial communities are essential for the proper development of gut-associated lymphoid tissue (GALT), production of mucosal IgA, and establishing T helper 1 (Th1) and T helper 2 (Th2) cells balance [68]. As the microbiota can shape the immune system, an altered intestinal microenvironment could contribute to immune-related diseases such as allergies and autoimmune disorders (IBD, type 1 diabetes). 

Moreover, emerging evidence suggests a bidirectional communication between the gut and the brain, called the microbiota-gut–brain axis. Gut bacteria were shown to influence neurodevelopment, and behavioral and cognitive functions [69]. Disruptions of the microbiota-gut–brain axis have been found in various neuropsychiatric disorders such as autism spectrum disorder, depression, dementia, schizophrenia, Alzheimer’s, and Parkinson’s disease [70,71,72].

Microbiota alteration, known as dysbiosis, has been involved in various diseases. An impaired intestinal flora has been proven to represent a developmental factor in obesity and metabolic syndrome, leading to type 2 diabetes and various cardiovascular impairments [73].

Thus, maintaining a proper symbiotic bacterial community is of paramount importance for the host health. Microbiome-targeted interventions, particularly in diseases that currently lack a causal treatment, could provide a starting point for a new class of biomarkers and therapies. 

## 4. Capsaicin and Microbiota Crosstalk

### 4.1. Modulation of the Gut Microbiota by Capsaicin

There is mounting evidence suggesting that CAP could influence the composition, abundance, and function of the intestinal microbial microenvironment. CAP interaction with the gut microbiota population is facilitated by its high concentration in the intestinal lumen before absorption, reaching levels between 500 and 1000 μM [9].

*Faecalibacterium prausnitzii* is an anaerobic bacterium (phylum *Firmicutes*) and the most important symbiotic component of the human gut microbiome [74,75]. It is considered a bioindicator of human health, being negatively associated with IBD, immunity, obesity, diabetes, asthma, major depressive disorder, and colorectal cancer [76,77,78,79,80,81,82,83]. In mice treated with CAP by intragastric perfusion (8 mg/kg/day) for one week, *Faecalibacterium* that was initially absent was detected in the CAP-treated group but not in controls, and increased gradually with intragastric perfusion in male but not in female mice [21]. Moreover, a short-term and high-dose CAP-enriched diet (10 mg/day, for two weeks) has been proven to increase bacteria abundance in healthy humans [84]. This suggests that CAP is a determinant factor for *Faecalibacterium’s* presence in the intestinal flora.

*Roseburia* is another important Gram-positive anaerobic bacteria (phylum *Firmicutes*) that inhabits the human intestines. Stimulation of this species proliferation has been associated with a reduction in glucose intolerance and with body-weight loss in mice [85]. CAP, administered by intragastric perfusion for one week in a dose of 8 mg/kg/day, enhanced the *Roseburia* gut content of male but not of female mice, suggesting a sex-based difference in the effect of CAP [21]. Another study conducted in spontaneous obese diabetic male mice with genetic mutations (*ob*/*ob* mice) showed that dietary CAP administered in low doses (0.01%), or high doses (0.02%), and for a longer time (six weeks) led to an increase in *Roseburia* abundance in the intestine [86].

Moreover, Song et al. demonstrated that CAP administration augmented the *Firmicutes/Bacteroidetes* ratio at the phylum level and decreased *Bacteroides* and *Parabacteroides* gut content at the genus level [86]. Similar findings regarding the *Firmicutes/Bacteroidetes* ratio have been reported in humans. Both low and high concentrations of dietary CAP (respectively 5 and 10 mg/day) administered for each one for 2 weeks in healthy subjects led to an increase in this ratio of the overall intestinal flora balance [84]. It has also been shown that CAP actions may depend on host gut enterotype, with more benefits obtained for enterotype 1 (*Bacteroides* enterotype) than for enterotype 2 (*Prevotella* enterotype). The author suggested that personalized nutrition guidance with dietary CAP may be considered, depending on intestinal microbiota stratification [84].

*Bacteroides* species are involved in reducing gut host complex molecules into simpler ones [87,88]. *Bacteroides* population abundance has been found to decrease in male mice subjected to CAP intragastric perfusion (8 mg/kg/day) for one week, but not in females, suggesting a sex-dependent manner of CAP action [21]. In another experimental setting, a low dose of dietary CAP (2 mg/kg) administered every two days for 12 weeks restored the decreased level of *Bacteroides Prevotella* abundance to control level, in male mice fed with a high-fat diet (HFD) [89]. An in vitro study showed that CAP and several other phytochemicals exhibit inhibitory activity against *Prevotella bryantii* B14 (CAP concentration 0.33 mM) and *Bacteroides fragilis* 25285 (CAP concentration 0.33 mM), as representatives of the *Bacteroidetes*, respectively, against *Acetoanaerobium sticklandii* SR (*Clostridium*) (CAP concentration 0.33 mM) and *Clostridioides difficile* 9689 (CAP concentration 3.27 mM), as species of phylum *Firmicutes* [90]. The author suggested that these phytochemicals could impact the normal gut microbiota in a manner quite similar to clinical antibiotics.

*Lactobacillus* is a typical probiotic bacteria belonging to phylum *Firmicutes* and is important for the homeostasis of immune cells and intestinal host health [91]. *Lactobacillus acidophilus* species is able to increase the number of beneficial bacteria to the detriment of pathogenic ones in the intestine [92]. *Lactobacillus* has been reported to increase in mice fed with HFD and treated with CAP (2 mg/kg CAP every two days, for 12 weeks), compared with mice fed with HFD alone [89]. CAP has also been proven to increase L-lactate in bacterial culture, by enhancing the metabolic activity of *Lactobacillus acidophilus* [93]. In another study conducted in spontaneous diabetic male mice with a genetic mutation (*db/db*, leptin receptor-deficient diabetic mice), a CAP-enriched diet (at a low dose of 0.01%, for 8 weeks) mitigated insulin resistance and improved glucose homeostasis, an effect that was associated with the CAP-induced repression of the increased abundance of the genus *Lactobacillus* [94].

The effect of diet on the microbiome has been proven to depend on the host genotype. When different pepper-containing diets containing carotenoids and phenolic compounds have been administered in *Drosophila melanogaster* with three genetic backgrounds, an enhancement of *Lactobacillaceae* and *Acetobacteraceae* abundance has been evidenced [95].

Regarding the underlying mechanisms by which CAP acts on gut microbiota, they can be divided into direct (TRPV1-dependent) and indirect (TRPV1-independent effects). As previously mentioned, CAP represents a highly potent agonist of the TRPV1 channel, and this action probably underlies its main effect on gut microbiota. Intestines are richly innervated by afferent sensory nerves expressing TRPV1 channels, and their activation elicited an important role in the gut function regulation [96,97,98]. As a consequence of TRPV1 channel stimulation, dietary CAP could change gut excitability and sensitivity, and induce a local release of neuropeptides, such as substance P, or calcitonin-related peptide [96,97,98,99]. These neuropeptides can secondarily modulate gut microbiota composition and structure by modifying the inflammatory and immune conditions in the gut environment [86]. 

Studies in TRPV1 knockout (KO) mice (based on TRPV1 deletion) revealed distinct changes in gut microbiota composition and abundance. A reduced α-diversity was reported in KO mice, when compared to wild-type (WT) counterparts, as well as a β-diversity change by clustering several gut microbiota populations. At the phylum level, the *Firmicutes/Bacteroidetes* ratio diminished in KO mice, by reducing *Firmicutes* and enhancing *Bacteroidetes* abundance [100,101]. Moreover, ablation of TRPV1 neurons by the potent agonist resiniferatoxin induced a severe mucus level reduction, caused by the downregulation of multiple associated genes, and a reduction in the abundance of lactic acid-producing bacteria [101]. Thus, TRPV1 may be essential for proper mucin production and for the preservation of a healthy gut bacterial population. 

On the other hand, CAP was also shown to influence gut microbiota regardless of TRPV1 channel activation. In a recent study, CAP intragastrical administration at a low dose of 2 mg/kg for 12 weeks was shown to increase the abundance of several gut bacterial populations (*Akkermansia, Bacteroides, Prevotella, Odoribacter, Coprococcus, Allobaculum*, and *S24_7* family), and to decrease the abundance of others (*Desulfovibrio, Helicobacter, Escherichia*, and *Sutterella*) in WT female mice, but also in KO counterparts, all fed with a HFD [102]. 

Thus, CAP modulatory effects on gut microbiota are mediated by both dependent and independent mechanisms of TRPV1 channel activation. However, data are insufficient to draw a proper conclusion and more molecular studies are needed to elucidate the underlying subtle mechanisms of gut–capsaicin interaction.

### 4.2. Microbiota and the Effect of Capsaicin on Glucose Homeostasis

**Figure 1:**
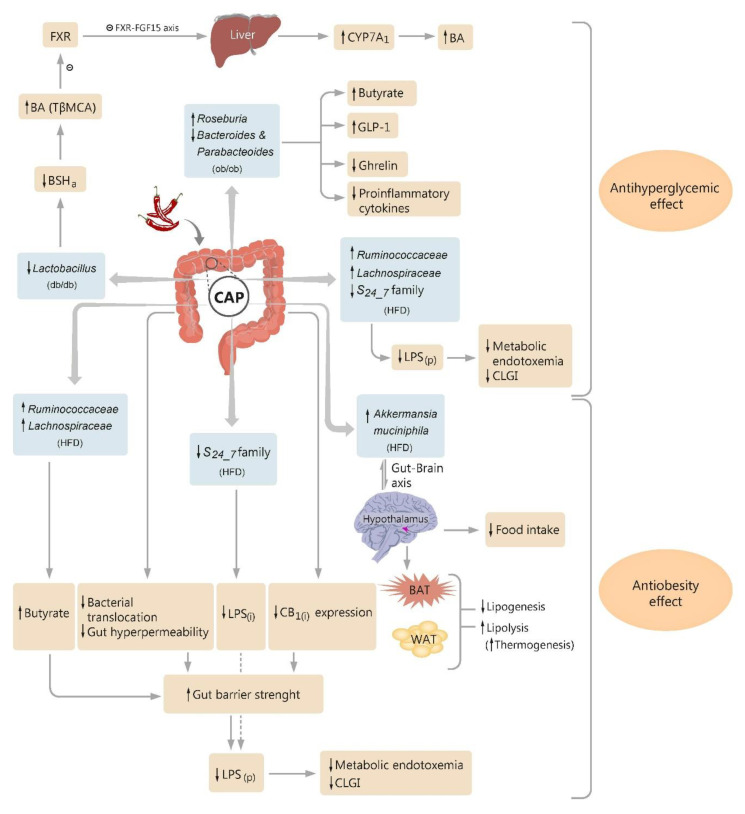
Diagram illustrating the proposed pathways by which dietary capsaicin (CAP) influences the glucose homeostasis and obesity, through modulatory action on intestinal microbiota. CAP decreases *Lactobacillus* abundance in type 2 diabetic mice (*db/db*), which reduces the bile salt hydrolase activity (BSH_a_), increases the levels of conjugated bile acids (BA) in the gut, and especially tauro-β-muricholic acid (TβMCA), an antagonist of farnesoid X receptor (FXR). A change in FXR signaling occurs and also a suppression in enterohepatic FXR-FGF15 axis (FGF15—fibroblast growth factor 15), leading to an upregulation of the cholesterol 7α-hydroxylase (CYP7A_1_) expression and enhancement in hepatic BA synthesis. CAP increases *Roseburia* and suppresses *Bacteroides* and *Parabacteroides* abundances in obese diabetic mice (*ob/ob*), followed by an increase in fecal butyrate level and plasma glucagon-like peptide-1 (GLP-1), and a reduction in plasma total ghrelin and proinflammatory cytokines. CAP exerts anti-obesity effects in high-fat diet (HFD)-fed mice by modulating the gut–brain (hypothalamus) axis, finally targeting brown adipose tissue (BAT), white adipose tissue (WAT), and mice food intake. CAP diminishes the abundance of Gram-negative pathogens able to secrete LPS_(i)_ (intestinal bacterial lipopolysaccharide), such as *S24_7* family members, and increases the butyrogenic bacteria abundance (e.g., *Ruminococcaceae* and *Lachnospiraceae*), and consequently the fecal butyrate, in HFD mice. CAP attenuates the increased gut permeability and bacterial translocation, and suppresses the intestinal cannabinoid receptor type 1 (CB_1(i)_) expression, in HFD mice. By these pathways, CAP increases gut barrier strength in these obese mice, which alongside the reduction in high levels of LPS_(i)_ generated by altered intestinal flora, results in a reduction in high levels of plasma-circulating LPS (LPS_(p)_), a reduction in metabolic endotoxemia, and alleviation of the chronic low-grade inflammation (CLGI). Other symbols: ↑- up regulation, ↓- down-regulation.

CAP has an acknowledged role in glucose homeostasis generally via the TRPV1 channel. This further enhances calcium influx and secretion of GLP-1 (glucagon-like peptide-1) from the intestinal cells of mice and humans [84,103]. GLP-1 and GIP (gastric inhibitory polypeptide) are known to lower blood glucose by enhancing insulin secretion and inhibiting glucagon release [104]. However, this is not the only mechanism through which CAP exerts its action. CAP is also able to modulate microbiota composition and abundance, therefore mitigating the impaired glucose tolerance and insulin resistance (Figure 1). 

It has been shown that CAP, at a low dose of 0.01% supplemented in the diet of type 2 diabetic *db/db* mice for 8 weeks, resulted in microbiota remodeling, by significantly inhibiting the increase in *Lactobacillus abundance* induced by diabetes. This led to a decrease in the bile salt hydrolase activity (BSH_a_), which then increased the levels of conjugated bile acids (BA) in the gut, and especially TβMCA (tauro-β-muricholic acid), a natural antagonist of farnesoid X receptor (FXR). CAP intervention increased the ratio of conjugated/unconjugated BA (in accordance with the increased level of BSH_a_), most likely due to a marked increase in TβMCA (1.5-fold). We wonder what is the mechanism of TβMCA augmentation, especially as it appears to have the largest variation compared to all other tested conjugated-BA [94]. Probably, because taurine, a semi-essential amino acid, which has been shown to have numerous beneficial effects in the human body [105,106,107,108], also targets the intestine and it is known to regulate gut microbiota by inhibiting harmful bacteria, reducing bacterial lipopolysaccharide (LPS) level, and accelerating short-chain fatty acids (SCFA) production [109,110]. Moreover, homotaurine stimulates GABA receptors in capsaicin-sensitive sensory neurons [111]; taurine-derived bile salts interfere with gastric mucosal blood flow and secretion, by modulating these sensory neurons [112,113,114]; and CAP infusion in the extracellular fluid increases taurine concentration [115]. In the comprehensive study of Hui et al., Pearson analysis further revealed a negative correlation between TβMCA and *Lactobacillus* abundance. Consequently, an inhibition of enterohepatic FXR-fibroblast growth factor 15 (FGF15) signaling occurred, followed by the upregulation of the cholesterol 7α-hydroxylase (CYP7A_1_) expression and hepatic BA synthesis (Figure 1). Changes in FXR intestinal signaling improved glucose homeostasis and alleviated insulin resistance by increasing hepatic glycogen storage and reducing hepatic gluconeogenesis [94]. This action might be partly explained by a reduction in glucose 6-phosphate absorption, or a decrease in ceramide levels, which diminishes hepatic pyruvate carboxylase activities and mitochondrial acetyl-CoA [82,116,117]. Moreover, antibiotic therapy initiated in the next experiment of this study depleted gut microbiota and abolished CAP benefits on BA metabolism and glucose homeostasis. Hui et al. concluded that CAP administration in *db/db* diabetic mice resulted in a suppression of enterohepatic FXR-FGF15 axis by preventing the increase in abundance of *Lactobacillus* genus, which further induced an enhanced BA levels, improved glucose metabolism, and increased insulin sensitivity [94]. Therefore, modulation of this physiopathological axis may represent a novel approach in future therapeutic strategies aiming to delay type 2 diabetes mellitus progression.

Song and collaborators demonstrated that dietary CAP enhanced the abundance of *Roseburia* and suppressed the abundances of *Bacteroides* and *Parabacteroides* in a genetic model of mice simulating human obesity-related type 2 diabetes (see previous section). These two bacterial changes at the genus level were respectively negatively and positively correlated to the fasting plasma glucose and the area under the curve of the oral glucose tolerance test. Moreover, both low- and high-concentration CAP diets (respectively 0.01% and 0.02%) raised the fecal butyrate level, as well as plasma total GLP-1, and diminished plasma total ghrelin, IL-1b, IL-6, and TNF-a levels. Thus, the improvement in glucose homeostasis induced by dietary CAP seems to be associated with a change in microbiota composition and may be ascribed to the rise in fecal butyrate, regulation of gastrointestinal hormones (total GLP-1 and ghrelin), and inhibition of the proinflammatory cytokines (Figure 1). Additionally, it was speculated that CAP activation of gut TRPV1 can lead to local release of neuropeptides, such as substance P or calcitonin gene-related peptide, which are able to regulate the gut microbiota content. Overall, it was concluded that the antihyperglycemic role of CAP, mediated by gut microbiota change, may provide a novel insight into the additional therapeutic tools useful in combating the impaired glucose metabolism [86].

Accumulating evidence suggests that reduced plasma levels of bacterial lipopolysaccharide (LPS) may represent a potent strategy in combating metabolic diseases [118,119]. Recently, an exhaustive study demonstrated that CAP administered in diet at a low dose of 0.01%, for 12 weeks, was able to reduce the abundance of Gram-negative LPS-producing bacteria, particularly members of the *S24_7* family, in mice fed with a HFD. CAP treatment also reduced the passage of LPS to systemic circulation and plasma levels of LPS, diminished the levels of circulant proinflammatory cytokines (TNF-α, IL-1β, IL-6), and alleviated insulin resistance (glucose intolerance, measured by oral glucose tolerance tests) [120]. It is well known that excessive LPS production induced by dysbiosis is linked to metabolic endotoxemia and chronic systemic low-grade inflammation (CLGI) [120]. CLGI further promotes pancreatic beta cells injury, disturbance of insulin action, and induces glucose intolerance in obesity [121]. So, it has been suggested that CAP intervention can play an important role in improving insulin resistance, by beneficially altering the gut flora of HFD-fed obese mice [120].

Dihydrocapsiate (DHC), a nonpungent capsaicin alternative, readily available as a synthetic derivative used to enrich a wide range of food products, shares similar metabolic benefits. DHC orally administered in HFD-fed mice in a low dose of 2 mg/kg, or high dose of 10 mg/kg, for 12 weeks altered the gut bacterial abundance, improved gut morphology, normalized the expression of genes regulating glucose metabolism, lowered hepatic glucose levels, and improved serum insulin and glucose tolerance [122].

### 4.3. Microbiota and the Antiobesity Effect of Capsaicin

As obesity nowadays represents a disease with a pandemic spread that urgently requires alternative therapeutics, the ability of CAP to reduce body weight may be considered. A significant number of studies focused on CAP anti-obesity potency and various types of underlying mechanisms have been suggested. There is evidence that CAP reduces weight gain by activating the TRPV1 cation channel, subsequently enhancing BAT (brown adipose tissue) activity and inducing thermogenesis, increasing lipid oxidation and inhibiting adipogenesis in WAT (white adipose tissue), enhancing satiety and suppressing appetite in the hypothalamus, and last but not least, modulating the gastrointestinal function and gut microbiota [123,124,125,126,127]. However, a recent study reported that CAP is able to exert anti-obesity effects regardless of TRPV1 channel activation, as shown in TRPV1 KO mice. Intragastrical administration of CAP at a low dose of 2 mg/kg for 12 weeks resulted in a beneficial alteration of gut microbiota, increased SCFA level, and lower food intake and weight gain, in both WT and KO female mice fed with HFD [102].

*Akkermansia muciniphila*, a strictly anaerobic Gram-negative mucin-degrading bacteria from the human intestinal flora, is believed to exert an important role in combating obesity, diabetes mellitus, and asthma [81,128,129,130]. An inverse correlation between *Akkermansia* content and body-weight has been registered after 8 days of intragastric CAP perfusion (8 mg/kg/day) in mice, the effect of the dietary phytochemical on bacterial abundance being noted to be sex-sensitive [21]. In another study with a larger sample size and statistical power, CAP administration (at a low dose of 2 mg/kg) every two days, for 12 weeks in the diet of HFD-induced obese mice, resulted in a significant increase in *Akkermansia muciniphila* gut content. Corroborated with other CAP-induced effects (modulation of satiety-associated genotype, up-regulation of thermogenesis expression, increase in mitochondrial biogenesis potential in BAT, and induction of “browning” genotype in subcutaneous WAT), a novel putative anti-obesity mechanism exerted by dietary CAP has been suggested, involving a gut–brain (hypothalamus) and brain–adipose tissue axis [89] (Figure 1). Several years later, Baboota and colleagues resumed the previous experiments with chronically treated HFD-fed mice, this time using DHC, a capsinoid that has been shown to reduce body mass index and Lee’s obesity index in a dose-dependent manner (low dose of 2 mg/kg and high dose of 10 mg/kg, successively). DHC treatment also reduced at both doses the total SCFAs levels, this effect being attributed to specific microbial changes in *Firmicutes* and *Bacteroidetes* gut content. SCFAs result from intestinal fermentation of carbohydrates, which are then absorbed into the liver and converted into triacylglycerols. As SCFAs account for one per ten daily energy requirements in humans and have been noted to increase in overweight and obese people [131,132], their reduced level in this study was interpreted as a result of the energy-harvesting potential of altered intestinal microflora, induced by DHC intervention [122]. Increased gut content of *Akkermansia muciniphila* has also been noted in a recent study carried out in C57BL/6 male mice, fed with HFD and subjected to CAP treatment (a dietary low dose of 0.01%, for 9 weeks). CAP administration led to a reduced weight gain, an improvement in glucose tolerance, and an up-regulation of *Reg3g* (antimicrobial protein gene) and *Muc 2* (mucin 2 gene) expression in the intestine. It was concluded that the anti-obesity effect of CAP may be ascribed to a modest modulation of the gut microbiota [133].

On the other hand, there are data showing that CAP has no influence on obesity. Song et al. showed that even though both low or high doses of dietary CAP (0.01%, or 0.02%) manifested important effects on glucose homeostasis by remodeling gut microbiota, these failed to exert any inhibitory effects on obesity-related phenotypes, as they had no influence on adiposity index, body weight, and Lee’s obesity index, even if a decrease trend was observed [86]. However, this study found that CAP in both doses increased fecal butyrate, plasma total GLP 1, and decreased plasma levels of total ghrelin and proinflammatory cytokines. As these markers’ changes induced by gut microbiota regulation have been linked to obesity development [118,119,134,135,136,137,138], the findings of Song’ study are quite intriguing. The author explained the conflicting results related to glucose homeostasis and obesity by two reasons. First, CAP doses used in this study are probably not high enough to produce any effect on obesity, as the dietary dose should have exceeded 0.02%, and the feeding time ideally should have been longer than 6 weeks. Moreover, the CAP dose that inhibits obesity has been previously suggested to be higher than that required to improve glucose homeostasis [139]. Second, the anti-obesity effect of CAP might depend on the obese animal model used in the experiments. Studies on HFD mice constantly reported a beneficial influence of dietary CAP in regulating both glucose homeostasis and obesity [120,123,140,141]. Conversely, studies on genetic models of obesity and diabetes, such as those performed by Song et al. in *ob/ob* mice or by Okumura et al. in genetic *KK-Ay* mice (a diabetic strain developing type 2 diabetes with mild obesity, due to peripheral insulin insensitivity) [139], evidenced only antihyperglycemic effects, without changes in the obesity-related phenotype. Therefore, features and pathogenesis of obese-diabetic genetic models are likely to be different from those of dietary models of mice developing obesity and diabetes [86].

In another experiment conducted in a mice strain of genetic-induced diabetes, the *db/db* model, dietary CAP was found to exert important antihyperglycemic effects, mediated by microbiota regulation (study presented in the previous sections) [94]. No tests assessing CAP influence on obesity were performed in this study, but one can speculate that emerging findings may point to indirect effects on obesity. Hui et al. found that the CAP-induced reduction in increased *Lactobacillus* abundance in *db/db* mice was able to promote an FXR inactivation in ileum [94]. It has been previously reported that intestine-selective FXR inhibition can improve obesity, insulin resistance, and can attenuate nonalcoholic fatty liver disease in HFD mice, suggesting a possible therapeutic strategy in metabolic disorders [117]. Moreover, Hui and collaborators showed that CAP intervention prevented the excessive increase in *Firmicutes*, but exerted no action on the reduction in *Bacteroidetes* abundance, in *db/db* mice. These changes at the phylum level have been linked to obesity-driven dysbiosis [142,143,144], raising the hypothesis that CAP may act on obesity in *db/db* mice by also interfering with this pathway.

**Figure 2:**
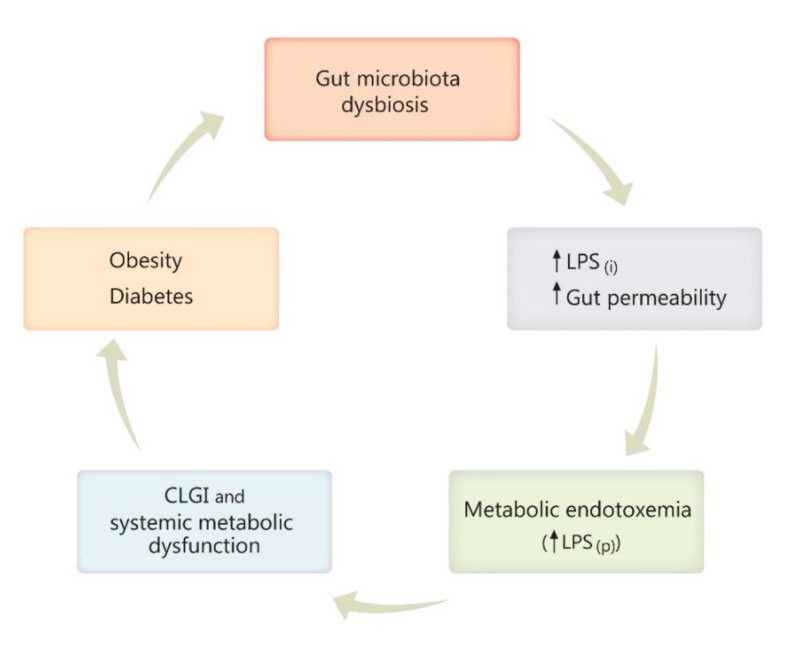
Diagram illustrating the vicious circle engaging gut microbiota dysbiosis, obesity, and diabetes. Gut dysbiosis, characterized by increased abundance of Gram-negative pathogens secreting bacterial lipopolysaccharide (LPS), leads to an increased gut permeability and increased intestinal LPS production (LPS_(i)_), favorizing LPS passage into the plasma (LPS_(p)_) and subsequent metabolic endotoxemia. Further, chronic low-grade inflammation (CLGI) and the accompanying systemic metabolic dysfunction contributes to body-weight gain, development of obesity, diabetes, and its possible complications that may arise, if the vicious circle is not interrupted. Other symbols: ↑- up regulation, ↓- down-regulation.

Gut dysbiosis has been repeatedly reported as one consequence of a high-fat diet, obesity, and diabetes, predisposing to inflammation, increased gut permeability, and enhancement of Gram-negative pathogens able to secrete LPS [118,119,145,146]. In the next stage of this histopathological chain, metabolic endotoxemia occurs through the passage of LPS to the systemic circulation, with subsequent inflammation [118,119]. Furthermore, chronic low-grade inflammation (CLGI) generates systemic metabolic dysfunction, which then contributes to body weight gain, development of obesity, diabetes, and their further complications that may arise [118,119,134,147,148], if the vicious circle is not interrupted (Figure 1 and Figure 2).

A very comprehensive study demonstrated that low doses of CAP intake (0.01% in the diet), for 12 weeks, downregulated the expression of gut tight junction proteins, diminished the increased intestinal permeability and bacterial translocation, and improved metabolic endotoxemia by alleviating the passage of LPS to the systemic circulation in wild-type HFD-fed mice [120] (Figure 1). CAP treatment also enhanced the butyrogenic bacteria abundance (*Ruminococcaceae* and *Lachnospiraceae*) and markedly increased the fecal butyrate, which is known for its capacity to strengthen the intestinal barrier, therefore alleviating metabolic endotoxemia [149]. Adipose tissue is the main tissue that releases CLGI markers in response to LPS. Thus, in the same experiment Kang et al. showed that CAP intervention can decrease the levels of systemic CLGI markers (IL-1β, IL-6, TNF-α) in HFD mice. Additionally, CAP was found to diminish the abundance of the LPS-producing *S24_7* family, downregulate the genes expression involved in LPS biosynthesis, and inhibit the intestinal cannabinoid receptor type 1 (CB1), which was reported to link metabolic endotoxemia to intestinal flora alteration [120,150,151]. By all these complex pathways, dietary CAP was able to improve insulin resistance and the obesity markers in this study, reducing fat pad weight, and weight gain (Figure 1). Last but not least, Kang and colleagues demonstrated that the anti-obesity effect of CAP is transferable to germ-free HFD-fed mice. While a cocktail of antibiotics (ampicillin, metronidazole, neomycin, and vancomycin) administered in these mice dropped out CAP-induced beneficial effects against HFD-induced obesity, further gut microbiota transplantation restored CAP protective phenotype. Taken together, all these findings made the author draw the conclusion that the intestinal microbiota act as a critical factor in the whole picture of CAP-induced anti-obesity effects, largely mediating these beneficial actions and even playing a causal role [120].

### 4.4. The Antimicrobial Property of Capsaicin

It is acknowledged that CAP has an important anti-bacterial function. The burden of increasing antibiotic resistance has determined interest in the anti-microbial properties of natural substances, such as CAP or capsinoids, to reduce the use of antibiotics [152].

The main factors responsible for the antibiotic resistance in all organisms are the bacterial multidrug efflux pumps. More than ten efflux pumps have been described for *Staphylococcus aureus* so far [153,154]. Mammalian P-glycoprotein inhibitors such as CAP and piperine inhibit these bacterial efflux pumps [155,156,157]. CAP (at increasing concentrations of 0.8 to 50 mg/L) has been proven to increase the susceptibility of *S. aureus* to antibiotics (such as ciprofloxacin), significantly decreasing the occurrence of ciprofloxacin-resistant mutants of the bacteria, and enhancing the post-antibiotic effect in a concentration-dependent way [156]. The plausible CAP antimicrobial mechanism seems to be similar to that exerted by the natural flavonoid Biochanin A, by inhibiting the activity of the multidrug efflux pump NorA [156].

CAP (at 2 mg/kg, orally) was also shown to decrease *Enterobacteriaceae* proportions in HFD-fed mice, reinforcing its antimicrobial property [89]. Moreover, CAP was found responsible for the inhibition of bacterial growth and the transfer of plasmids in *Escherichia coli*, a member of the *Enterobacteriaceae* family. Both CAP and gingerols, chemical relatives, were proven to pharmacologically inhibit the plasmid conjugation process [158]. On the other hand, one study showed that *Capsicum annuum* extract had no in vitro effect on *Escherichia coli* O157:H7 cultures [159], questioning the antibacterial activity of CAP against this bacterial family.

In vitro CAP treatment at a minimum inhibitory concentration of 64–128 μg/mL exerted a bactericidal action against *Streptococcus pyogenes* (Group A streptococci), a major human pathogen, in erythromycin-susceptible and erythromycin-resistant strains [22]. 

*Capsicum annuum* extract managed to inhibit bacterial growth of *Listeria monocytogenes* V7 cultures. Moreover, the extract fraction responsible for this effect was deciphered using spectral methods and primarily contained capsianoside derivatives [159].

Some strains of *Helicobacter pylori* are pathogenic to humans, causing peptic ulcers, gastritis, and gastric cancer [160]. CAP was proven to decrease the amount of *Helicobacter*, reducing the probability of diseases caused by these bacteria in a dose-dependent manner, at doses above 10 µL/mL, with a maximal effect at 50 µL/mL [21,161].

Although CAP antimicrobial activity has been investigated to some extent, findings come mostly from in vitro studies, which need to be substantiated in human clinical trials. 

### 4.5. Role of Capsaicin in Inflammatory Bowel Diseases

In IBDs, Crohn’s disease and ulcerative colitis, genetic factors combined with environmental triggers contribute to chronic inflammation and damage of the intestinal mucosa. Unfortunately, the conventional treatment of IBDs is burdened by side-effects being based on immunosuppressant and anti-inflammatory drugs. As animal model studies sustain the anti-inflammatory effect of CAP, could it find a place in IBD treatment? 

CAP is known to accelerate gut motility and to determine an anal burn sensation in healthy people [162]. Therefore, spices, including the bioactive ingredient CAP, are the most avoided foods in patients with IBD [163]. Patients with IBD present a high TRPV-1 immunoreactivity in colonic nerve fibers [164], in colonic epithelial mucosal cells, and in infiltrated inflammatory cells. This increased TRPV1 expression does not correlate with disease activity assessed through histological inflammation, inflammation biomarkers, and mucosal appearance at endoscopy [165]. Furthermore, immunohistochemistry of mucosal colonic biopsies collected from patients with IBD in remission revealed that abdominal pain severity was associated with increased TRPV1 colonic expression [166]. Thus, CAP may exacerbate abdominal pain in these patients, being an agonist of TRPV1 receptors. However, there is no direct evidence that CAP could make IBD symptoms worsen. On the contrary, CAP treatment could improve symptomatology [167]. A CAP-enriched diet (0.75 mg/day, 4 weeks) was shown to desensitize this receptor, causing it to be refractory alongside the nerve fiber [168], thus alleviating abdominal pain.

Assessment of stool samples from healthy subjects and patients suffering from IBDs revealed decreased microbiota diversity and a reduced microbiome, with 25% fewer genes in IBD patients compared to healthy individuals [52,169]. In addition, ileal mucosa fragments from Crohn’s disease patients showed a low abundance of *Faecalibacterium prausnitzii*, which has been associated with endoscopic relapse at 6 months [170]. *Faecalibacterium prausnitzii* is known to have anti-inflammatory properties, reducing the production of proinflammatory cytokines and increasing the secretion of anti-inflammatory cytokine IL-10 in peripheral blood mononuclear cell cultures and in an animal model of colitis, respectively [170]. Furthermore, Kawaguchi et al. study suggested that IBD patients have an altered immunological tolerance to food antigens similar to IL-10 knock-out mice via CD4+ T-cell hyperactivation [171]. Diets enriched in CAP could have a beneficial effect in Crohn’s disease, as they increase the *Firmicutes/Bacteroidetes* ratio and *Faecalibacterium* abundance [84], thus changing the immune balance to a more tolerogenic state for food antigens and commensal bacteria.

Several rodent models of colitis offered promising results regarding the mucosal protective effects of ingested CAP. Trinitrobenzene sulfonic acid-induced colonic ulcerations were partially prevented by topical CAP application (0.25 mL, 640 μM, colonic acute application through a canula) [172]. In dextran sulfate sodium-induced colitis, CAP ingestion (1–10 mg/kg, for 6 consecutive days) prevented the colonic mucosal damage [173]. High-dose CAP ingestion (10 mg/kg), 30 min before and 9 h after indomethacin subcutaneous injection, diminished the occurrence of small bowel ulcers in rats [174]. These benefits seem to be mediated through TRPV1 afferent sensory nerve endings. Sensory deafferentation by subcutaneous CAP injections (50 mg/kg, 3 consecutive days) induced a three-fold augmentation of the colonic damage caused by the application of acetic acid into the proximal colon [175]. Moreover, the destruction of CAP-sensitive sensory neurons in the gut mitigates the protective effects of a CAP-enriched diet in a dextran sulfate sodium-induced colitis model [173]. However, the pathophysiological mechanisms occurring in animal models may not overlap those underlying IBDs. Thus, further clinical studies would be required to prove the effectiveness of CAP in IBD patients.

The amount of CAP in the diet is also important, as high in vitro concentration (more than 100 μM) has been proven to increase intestinal permeability through a direct cytotoxic effect, and by increasing tight junctions’ permeability in intestinal epithelial cells monolayers [176]. On the other hand, a 0.01 g CAP/100 g diet administered in HFD-mice increased the intestinal barrier strength [120], suggesting a potential benefit in preventing endotoxemia associated with IBDs.

## 5. Capsaicin, a Spicy Molecule Entraining the Microbiome Function

It is obvious that the gut bacterial profile is strongly linked to diet. CAP, the major pungent component in red chili and a very popular worldwide phytochemical, displays modulatory effects on gut microbiota. Diets enriched with CAP and its derivatives have been proven to increase gut bacteria abundance by facilitating colonization with *Faecalibacterium prausnitzii* and *Roseburia*, which are important butyrate-producing bacteria required for the energy metabolism control, and for the commensal flora balance. On the other hand, CAP has been proven to decrease the abundance of LPS-producing Gram-negative bacteria, such as *S24_7* family members, to strengthen the intestinal barrier, therefore impeding LPS passage to systemic circulation. Moreover, CAP has also been shown to inhibit pathogenic bacteria growth by exhibiting a bactericidal effect, such as in the case of *Streptococcus pyogenes* and *Helicobacter pylori*. Unfortunately, the mechanisms by which CAP and capsinoids reshape the intestinal microbiota and change specific bacteria abundances are not completely elucidated.

The action of CAP on the gut content of a specific bacterial genus may occasionally seem to be capricious at first glance, some studies reporting positive and others negative influences. However, at closer inspection, CAP actually alleviates the gut abundance variations induced by different pathophysiological conditions, being able to even bring it back to normal. The heterogeneity of findings may also result from the large variability in the studies designs, the level of CAP concentration reached in the gut, or even from the species susceptibility to CAP interventions. When assessing the CAP–microbiota crosstalk, one should take into account that individuals have different genotypes and enterotypes, promoting specific responses to CAP-enriched diets. Personalized nutrition guidance with dietary CAP may then be considered.

Growing evidence links CAP and capsinoids dietary intake to improved obesity, glucose homeostasis, and insulin sensitivity. In the era of an intense worldwide-spread of these diseases, alternative therapeutics should be urgently considered. Recent studies have suggested several mechanisms by which CAP and its derivatives may act against these pathologies (Figure 1). As metabolic endotoxemia and associated CLGI have a pivotal role in obesity and diabetes pathogenesis, novel mechanisms of CAP influence have been proposed, involving the prevention of microbial dysbiosis and intestinal barrier dysfunction. CAP has also been shown to manifest anti-hyperglycemic and anti-obesity effects by modulating the gut–brain axis and inhibiting the entero-hepatic FXR-FGF15 axis. Not least, by enhancing the abundance of some gut butyrate-producing bacteria, by increasing the plasma level of glucagon-like peptide-1 (GLP-1), and by reducing plasma total ghrelin and circulant proinflammatory cytokines, CAP intervention brings new arguments to its putative role in combating obesity and diabetes. Even if several lines of evidence are likely to support the view that CAP treatment may function as a potent strategy for controlling metabolic diseases, underlying mechanisms by which CAP exerts such a complex influence requires further clarification. Moreover, the data come mostly from experimental studies, and there are many differences between humans and animals regarding interactions between CAP, intestinal flora, and FXR-FGF15 signaling, for instance. Therefore, directly extrapolating published animal findings to humans should be avoided. In the meantime, future randomized, placebo-controlled human trials to validate these data are warranted.

The same benefit of CAP in restoring the microbiota composition and abundance could be applied in IBDs, thus promoting a suppression of local inflammation. In Crohn’s disease particularly, dysbiosis characterized by low *Faecalibacterium prausnitzii* may be improved through CAP-enriched diets. Moreover, the traditional restriction for CAP use in IBDs is not evidence-based. The dosage of CAP in diets is probably one of the bias factors that promotes such a variability. It is well known that high doses of CAP could alter the intestinal barrier, while common doses decrease the permeability of the gut intestinal barrier, and this was proved in both in vitro and in vivo animal studies. Even if side-effects associated with CAP-enriched diets have been reported, such as those observed in oncological diseases, these complications are likely to be linked to the administration of high CAP doses, and/or for a longtime exposure. Furthermore, there are long-term prospective studies suggesting that spicy food consumption is associated with a lower risk of death. For certainty, much more experimental and clinical trials are needed before providing the optimal CAP dosage, adjustable according to individual enterotype and to diverse, subjacent pathological condition. 

Overall, a considerable body of evidence, mostly coming from animals, suggests that CAP and capsinoids exert multiple benefits on gut microbiota, involving various and complex mechanisms, targeting mainly metabolic and inflammatory diseases. The question that arises now is if this little, spicy molecule could be strong enough to strengthen the paradigm, and to show efficacity in combating pathologies considered nowadays global public health issues.
